# Internal inguinal hernia on the transplant side after kidney transplantation: a case report

**DOI:** 10.1186/s40792-015-0094-5

**Published:** 2015-10-17

**Authors:** Akihiro Kondo, Yuji Nishizawa, Shintaro Akamoto, Masao Fujiwara, Keiichi Okano, Yasuyuki Suzuki

**Affiliations:** Department of Gastroenterological Surgery, Faculty of Medicine, Kagawa University, 1750-1 Ikenobe, Miki-cho, Kita-gun, Kagawa 761-0793 Japan; Department of Colorectal Surgery, National Cancer Center Hospital East, 6-5-1, Kashiwanoha, Kashiwa-City, Chiba 277-8577 Japan

**Keywords:** Kidney transplantation, Inguinal hernia, Lichtenstein operation

## Abstract

The patient was a 52-year-old man who presented with right inguinal swelling and pain. He had undergone kidney transplantation in 2005 and bypass surgery using a vascular prosthesis from the left axillary artery to the bilateral femoral arteries in 2008. The vascular prosthesis had invaded the right inguinal canal ventrally. The transplanted ureter had a hazy appearance on a non-enhanced abdominal CT scan. A Lichtenstein operation was performed under a diagnosis of inguinal hernia. A skin incision with pulling of tissue and subcutaneous fat was devised to avoid exposure of the vascular prosthesis. The inguinal canal and spermatic cord were found to have coalesced. The hernia was diagnosed as a supravesical hernia, class II-1. This case shows that a Lichtenstein operation is a suitable procedure for avoidance of damage to the transplanted ureter in treatment of a transplant-side inguinal hernia in a kidney transplant recipient.

## Background

The increased frequency of living-donor or brain-dead kidney transplantation has led to observation of rare post-transplant complications, including kidney transplant-associated inguinal hernia. Here, we describe a case in which internal inguinal hernia developed on the transplant side 7 years after living-donor kidney transplantation. We also provide a literature review of this condition.

This literature had been published in Journal of Japan Surgical Association 2014, volume 75(3), 841–844 pages in Japanese [1].

## Case presentation

The patient was a 52-year-old man with a chief complaint of swelling and pain in the right inguinal region, which he had experienced since February 2012. His medical history included a living-donor kidney transplantation in the right iliac fossa for chronic renal failure in 2005. He had also undergone artificial graft bypass surgery from the right axillary artery to the bilateral femoral arteries for treatment of bilateral arteriosclerosis obliterans (ASO) in 2008. He had no particular family medical history.

**Fig. 1:**
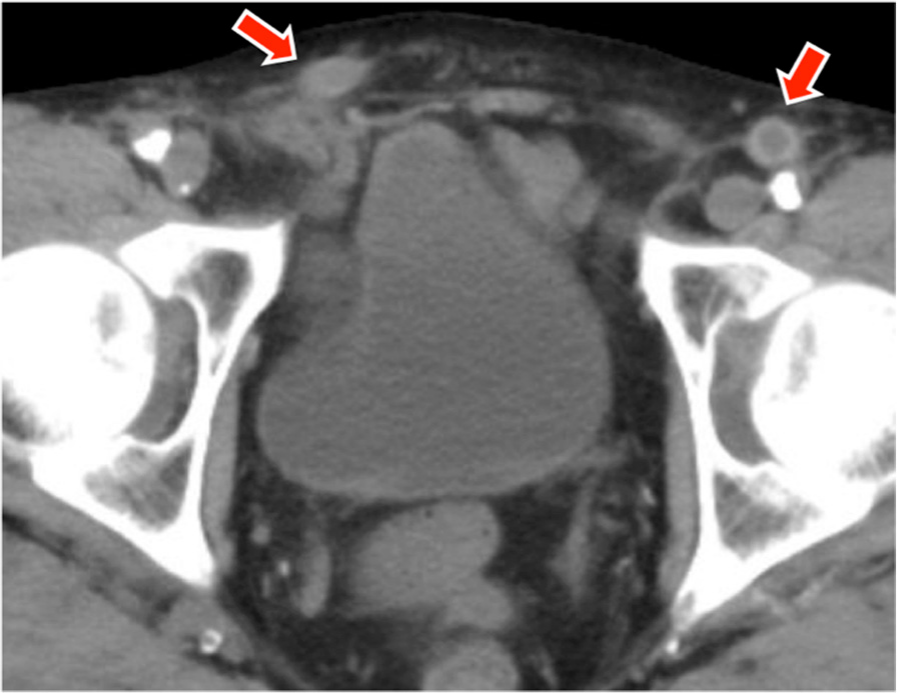
Non-enhanced abdominal CT scan (axial) showing the vascular prosthesis bilaterally (*arrow*), one on the *right* side positioned ventrally in the right inguinal canal

**Fig. 2:**
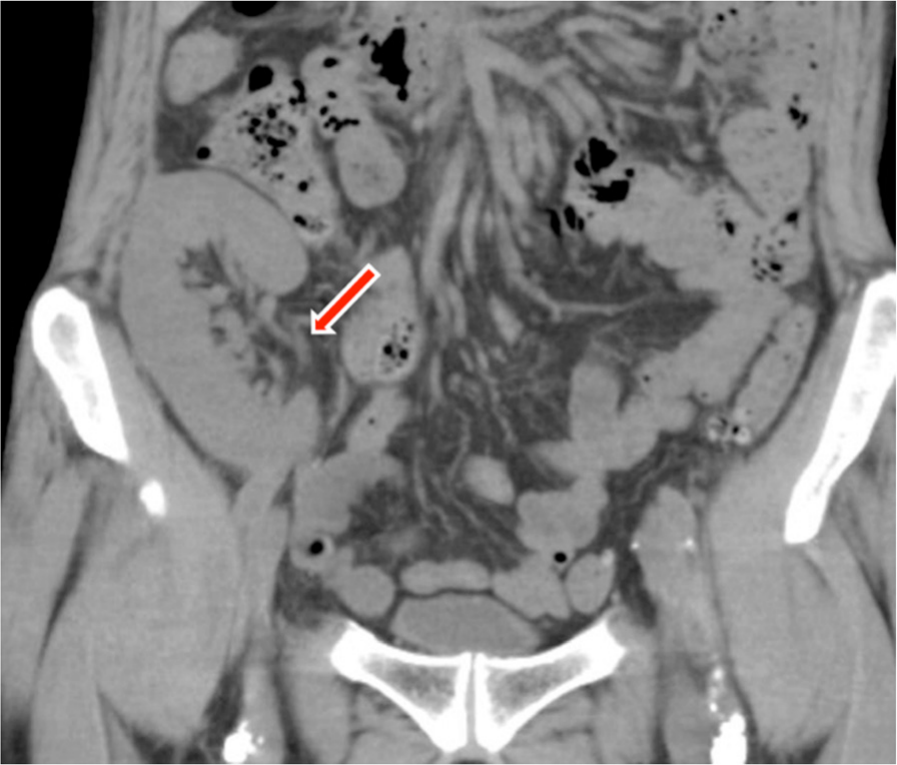
Non-enhanced abdominal CT scan (coronal) shows that the location of the transplanted ureter is unclear (*arrow*)

At the first examination, physical findings were height 179 cm and body weight 60 kg. Hernia in the right inguinal region was noted in a standing position or while defecation. Blood chemistry findings included urea nitrogen, 40.8 mg/dl; creatinine, 2.44; potassium, 5.4 mmol/l; and hemoglobin, 7.6 g/dl; all of which indicated renal dysfunction and anemia. On plain abdominal CT, a subcutaneous vascular prosthesis was present on the ventral side of the right inguinal canal (Fig. 1). The transplanted kidney was observed under the right ilium, but the location of the transplanted ureter was unclear (Fig. 2).

**Fig. 3:**
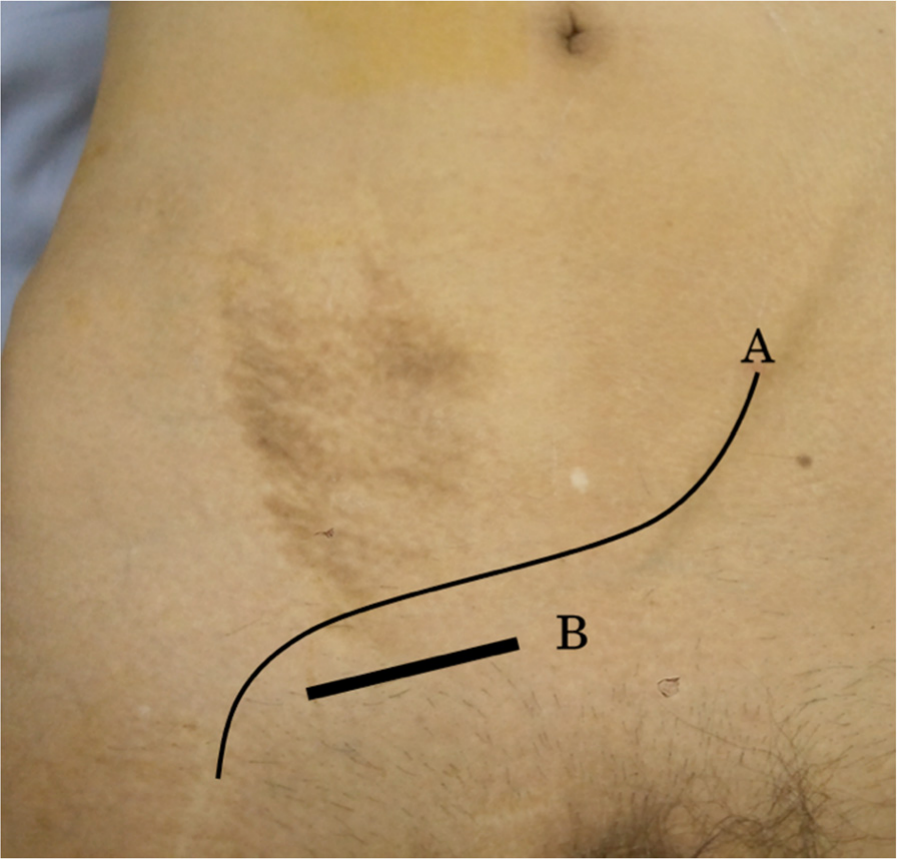
Preoperative physical findings. *A* Vascular prosthesis, *B* skin incision

**Fig. 4:**
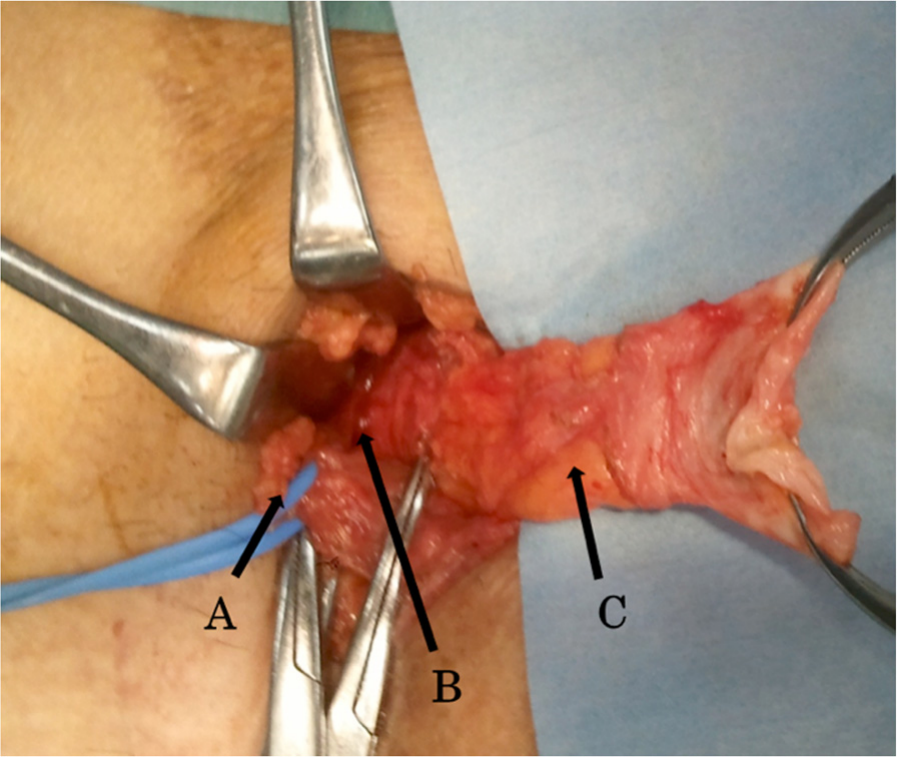
Intraoperative findings. *A* Spermatic cord, *B* hernia orifice, *C* hernia sac

Since the position of the transplanted ureter was uncertain, a right ureteral stent was placed before surgery. A skin incision was made at the lower margin of the vascular prosthesis palpable on the caudal side of the right inguinal canal, and the tissue was pulled with subcutaneous fat while avoiding exposure of the vascular prosthesis (Fig. 3). Mild adhesion of the inguinal canal around the spermatic cord was noted. Since a hernia orifice was palpated on the vesical side of the posterior wall of the inguinal canal, the patient was diagnosed with right internal inguinal hernia (supravesical hernia), class II-1 (Fig. 4). After dissecting the hernia sac, radical surgery for hernia was performed using the Lichtenstein method, which a single polypropylene mesh was fixed on the posterior wall of the inguinal canal. The stent placed in the right ureter was not palpated during surgery. The postoperative course was uneventful, and the patient was discharged 4 days after surgery. No recurrence of hernia or complication has subsequently occurred.

### Discussion

Inguinal hernia is encountered frequently in routine medical practice. The number of kidney transplantations has increased yearly in Japan, with 1610 procedures performed in 2012 alone, based on data from the Japanese Society for Transplantation. However, it is rare to encounter inguinal hernia developing on the transplant side after kidney transplantation, and this condition has to be treated at institutions certified for kidney transplantation.

Hernia occurred on the kidney transplant side in our patient and additionally, a vascular prosthesis had passed through under the skin on the ventral side of the inguinal canal. A skin incision was made on the caudal side in parallel to the vascular prosthesis, and the surgical field was maintained by pulling the tissue with attached subcutaneous fat to avoid exposure of and damage to the vascular prosthesis. Mild adhesion of the inguinal canal around the spermatic cord was noted and may have been due to the surgery performed for kidney transplantation, but dissection was relatively easy. The transplanted ureter, in which a stent had been placed, was not detected during surgery.

Twelve case reports about inguinal hernia after kidney transplantation has been reported so far in PubMed research (Table 1) [2–13]. Some literatures reported serious intraoperative accidents or postoperative complications in surgery for inguinal hernia associated with kidney transplantation, including unrecognized ligation of transplanted ureter [2], injury to the urinary bladder [3], transplanted ureter necrosis caused by mesh plug [14, 15], and transplanted ureteral obstruction caused by transabdominal preperitoneal repair (TAPP) [4]. But then, Koizumi et al. [16] were able to avoid complications involving the transplanted ureter by performing radical surgery for hernia using the Lichtenstein operation.

**Table 1:** Case reports of inguinal hernia after kidney transplantation

Author	Year	Age/gender	Post-kidney transplantation (years)	Hernia content	Operation methods	Morbidity
Selman [2]	1985	58/M	12	Transplanted ureter	McVay	Ureteric stenosis
Kobayashi [3]	2000	39/M	4	N/A	McVay	Urinary bladder injury
Sanchez [5]	2005	70/M	5	Transplanted ureter	Lichtenstein	None
Furtado [6]	2006	44/M	12	Transplanted ureter	N/A	None
Verbeeck [7]	2007	75/M	11	Transplanted ureter	N/A	None
Ingber [8]	2007	72/M	12	Transplanted ureter	Polypropylene mesh use	None
Otani [9]	2008	53/M	9	Transplanted ureter	N/A	None
Azhar [10]	2009	76/M	20	Transplanted ureter	N/A	None
Odisho [11]	2010	58/M	15	Transplanted ureter	Lichtenstein	None
Pourafkari [12]	2012	50/M	12	Transplanted ureter	No operation	Death
Tse [4]	2013	57/M	3	N/A	TAPP	Ureteric stenosis
Vyas [13]	2014	32/M	7	Transplanted ureter and bladder	N/A	None
Our case		52/M	7	N/A	Lichtenstein	None

*N/A* = not available

Lichtenstein et al. [17] first described the Lichtenstein operation for inguinal hernia. In this tension-free surgery, a monofilament polypropylene mesh is inserted into the posterior wall of the inguinal canal. The approaches which insert an underlay mesh into the anterior peritoneal cavity, such as Prolene Hernia System (PHS), direct Kugel methods, and mesh plug methods, may damage a transplanted ureter present in this lesion. In contrast, the Lichtenstein operation does not dissect the anterior peritoneal cavity and, thus, is a most suitable method to prevent complications involving the transplanted organs.

Patients after kidney transplantation take immunosuppressants for a long period, and infectious complications caused by the use of mesh may be a concern. Catena et al. [18] found that the Lichtenstein operation used the porcine small intestine submucosa as a mesh was safe for immunosuppressed patients, but suggested that nonabsorbable polypropylene prostheses is a well-known risk of infection. Since there is a problem of surgical site infection after the mesh repair for the post-transplant patients, it can be considered about optional methods that need no mesh, such as traditional Bassini’s or McVay’s method and Shouldice repair [19] that is recommended as “tissue repair method”.

There have also been 10 case reports (PubMed research) about transplanted ureteral obstruction caused by the inguinal hernia incarceration [5–13], so we need to attend the incidence of inguinal hernia after kidney transplantation. But we were unable to find studies of the relationship between kidney transplantation and the occurrence of inguinal hernia. There has been the need of amassed research evidence of a number of these cases.

## Conclusion

The case reported here illustrates the importance of protection of the transplanted ureter in the treatment of inguinal hernia on the transplant side in a kidney transplant recipient and shows that the Lichtenstein operation is a safe surgical procedure in such cases.

## Consent

Written informed consent was obtained from the patient for publication of this case report and any accompanying images. A copy of the written consent is available for review by the Editor-in-Chief of this journal.

This case report is being approved by the institutional ethical review board of Kagawa University Hospital.
